# Aberrant host immune response induced by highly virulent PRRSV identified by digital gene expression tag profiling

**DOI:** 10.1186/1471-2164-11-544

**Published:** 2010-10-07

**Authors:** Shuqi Xiao, Delin Mo, Qiwei Wang, Jianyu Jia, Limei Qin, Xiangchun Yu, Yuna Niu, Xiao Zhao, Xiaohong Liu, Yaosheng Chen

**Affiliations:** 1 State Key Laboratory of Biocontrol, School of Life Sciences, Sun Yat-sen University, Guangzhou, China

## Abstract

**Background:**

There was a large scale outbreak of the highly pathogenic porcine reproductive and respiratory syndrome (PRRS) in China and Vietnam during 2006 and 2007 that resulted in unusually high morbidity and mortality among pigs of all ages. The mechanisms underlying the molecular pathogenesis of the highly virulent PRRS virus (H-PRRSV) remains unknown. Therefore, the relationship between pulmonary gene expression profiles after H-PRRSV infection and infection pathology were analyzed in this study using high-throughput deep sequencing and histopathology.

**Results:**

H-PRRSV infection resulted in severe lung pathology. The results indicate that aberrant host innate immune responses to H-PRRSV and induction of an anti-apoptotic state could be responsible for the aggressive replication and dissemination of H-PRRSV. Prolific rapid replication of H-PRRSV could have triggered aberrant sustained expression of pro-inflammatory cytokines and chemokines leading to a markedly robust inflammatory response compounded by significant cell death and increased oxidative damage. The end result was severe tissue damage and high pathogenicity.

**Conclusions:**

The systems analysis utilized in this study provides a comprehensive basis for better understanding the pathogenesis of H-PRRSV. Furthermore, it allows the genetic components involved in H-PRRSV resistance/susceptibility in swine populations to be identified.

## Background

In 2006 there was an unparalleled large-scale outbreak of so-called 'high fever' disease in many areas of China that affected more than 2,000,000 pigs. There were approximately 400,000 fatal cases in 2006 and 243,000 fatalities in 2007, leading to concerns within the global swine industry and in relation to public health [1]. In March 2007 the disease was identified in the Hai Duong Province of Vietnam and it spread country-wide affecting more than 65,000 pigs [2]. The outbreaks caused extensive concern worldwide [3]. Affected pigs of all ages presented with clinical signs including continuous high fever (40.5°C-42°C), depression, anorexia, dyspnoea, reddening of the skin, edema of the eyelids, conjunctivitis, mild diarrhea, shivering, lamping, and unusually high morbidity (50%-100%) and mortality (20%-100%). Studies demonstrated that highly virulent porcine reproductive and respiratory syndrome virus (H-PRRSV) was the major causative pathogen of the so-called "high fever" disease. Genetic analysis indicated that the H-PRRSVs isolated from China and Vietnam shared a discontinuous deletion of 30 aa in non-structural protein 2 (NSP2), as compared with the North American type PRRSV strains (NA PRRSV). However, the mechanisms underlying the molecular pathogenesis of the H-PRRSV that emerged in China and Vietnam have not been elucidated.

Preliminary results indicated that PRRSV modulates the host immune responses and alters host gene expression. PRRSV infection up-regulated expression of mRNA for interleukin-10 (IL10), interferon gamma (IFN-γ), tumor necrosis factor-alpha (TNF-α), myxovirus resistance 1 (MX1), ubiquitin specific proteases (USP) and toll-like receptors (TLR), and inhibited expression of type I interferons [4-7]. A study concerning the genome-wide transcriptional response of primary alveolar macrophages (PAMs) following infection with the Lelystad PRRSV strain (European type, EU PRRSV) reported that the expression of beta interferon 1 (IFN-β1) was strongly up-regulated while expression of IL-10 and TNF-α was up-regulated slightly [8]. A further study concerning the effect of the VR-2332 PRRSV strain (NA PRRSV) on PAM function utilized serial analysis of gene expression and demonstrated that expression of MX1 and USP were significantly up-regulated 24 hours post-infection (h pi) [9]. These studies have provided a genome-wide gene expression profile of PAMs *in vitro *following infection with EU PRRSV or NA PRRSV. However, *in vitro *studies have significant limitations owing to disparities between the *in vitro *and *in vivo *environments. Therefore, characterization of host immune responses to PRRSV *in vivo *is required. PRRSV infection causes widespread apoptosis in pulmonary and lymphoid tissues of infected pigs [10], but the cause of the increased severity of the symptoms and the unusually high mortality of pigs infected with H-PRRSV (a mutant North American highly virulent porcine reproductive and respiratory syndrome virus) remains unknown.

High-throughput sequencing technology has been adapted for transcriptome analysis [11]. The technology developed by Illumina (formerly Solexa sequencing) [12], also referred to as Digital Gene Expression tag profiling (DGE) [13], allows millions of short RNAs and differentially expressed genes to be identified in a sample without the need for prior annotations. DGE has many advantages including greater sequencing depth, detection of unknown transcripts, practical implementation of digital tags, generation of absolute rather than relative gene expression measurements, detection of high levels of differential polyadenylation, detection of low-abundance transcripts and small changes in gene expression, that make it particularly attractive for measuring mRNA expression and identifying differentially expressed genes [14,15]. The hippocampal expression profiles of wild-type mice and δC-doublecortin-like kinase transgenic mice have been compared using Solexa sequencing technology [15], as have differences in gene expression between the liver and kidney [14]. Furthermore, the Illumina Genome Analyzer II platform has been used to perform DGE analysis of the zebra fish transcriptome response to mycobacterium infection [16]. However, DGE analysis has not been carried out on H-PRRSV infected pigs.

Herein histopathology, high-throughput deep sequencing and bioinformatics were utilized to analyze the relationship between pulmonary gene expression profiles after H-PRRSV infection and infection pathology. Comprehensive analysis of the global host response induced by H-PRRSV demonstrated that aggressive replication and dissemination of H-PRRSV resulted in an excessively vigorous immune and inflammatory response, contributing to severe tissue damage and high pathogenicity. This systems analysis could lead to a better understanding of the pathogenesis of H-PRRSV and to the identification of genetic components associated with H-PRRSV resistance/susceptibility in swine populations.

## Results

### Clinical and pathological features of H-PRRSV-infected pigs

H-PRRSV-infected pigs exhibited signs of 'high fever' disease within 3 days post-infection (d pi). They developed a persistent high fever of 41.0°C-41.7°C between 3d pi and 7d pi, presenting with reddening of the skin, dyspnoea, depression, anorexia, edema of the eyelids, conjunctivitis, mild diarrhea, rough hair coats, shivering and lamping.

Quantitative PCR (QPCR) demonstrated H-PRRSV virus 4 and 7d pi in all tissues tested, namely serum, heart, liver, spleen, lung, kidney, lymph and brain (Table S1 in Additional file 1). Moreover, the H-PRRSV virus was successfully recovered from each of the eight tissues investigated in the affected pigs. Higher levels of H-PRRSV virus were detected in serum, lung, spleen and lymph than in other tissues. Uninfected negative control (C) pigs had no clinical signs of disease, and H-PRRSV pathogens and viral re-isolates were absent.

**Figure 1:**
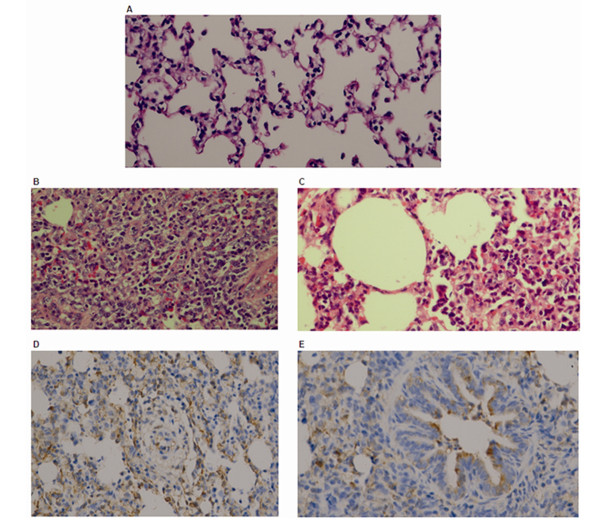
**Pathological examination of lungs infected with H-PRRSV on day 7 post-infection**. (A) Normal morphology of control pig lung; (B) robust interstitial pneumonia of the lung with marked thickening of alveolar septa accompanied by significant infiltration of immune cells; (C) emphysema in the lung; (D) most of the viral antigen (brown) was detected in alveolar cells; (E) and in bronchiolar epithelial cells in lesions.

Lungs of H-PRRSV-infected pigs presented with severe diffuse pulmonary consolidation lesions. Histopathological examination of H-PRRSV affected pigs demonstrated robust interstitial pneumonia and emphysema in the lungs with thickening of alveolar septa accompanied by extensive infiltration of immune cells (Figure 1B, C). The highest levels of viral antigen were detected in alveolar cells and bronchiolar epithelial cells of lesions (Figure 1D, E).

### Analysis of DGE libraries

Gene expression analysis was used to provide a global view of the host response in lungs of infected pigs in order to elucidate the aggressive virulence of H-PRRSV. Three porcine lung DGE libraries were sequenced from three C pigs (C), three pigs 96 h pi with H-PRRSV (H96) and three pigs 168 h pi with H-PRRSV (H168) using parallel sequencing on the Illumina platform. Major characteristics of these libraries are summarized in Table 1. An average of 5.9 million sequence tags per library was obtained, with 507109 distinct tag sequences. Prior to mapping these tag sequences to reference sequences adaptor tags, low quality tags and tags of copy number = 1 were filtered, producing an average of 5.6 million clean sequence tags per library, with 169,997 distinct clean tag sequences. The C library had the highest number of total sequence tags and distinct sequence tags, followed by the H168 and the H96 libraries. Furthermore, the C library had the highest ratio of number of distinct tags to total tags and the lowest percentage of distinct clean high copy number tags. More genes were detected in the C library than the other two libraries, and more transcripts were expressed at lower levels in the C library than in the others. Saturation analysis of the capacity of the libraries demonstrated that newly emerging distinct tags were gradually reduced with an increase in the number of total sequence tags. When the number of sequencing tags reached 3 million, library capacity approached saturation (Figure S1 in Additional file 1).

**Table 1:** Major characteristics of DGE libraries

	C		H96		H168	
			
	Distinct Tag	Total Tag	Distinct Tag	Total Tag	Distinct Tag	Total Tag
Raw Data	789417	7767249	366733	4515335	365178	5501868
Low Quality Tag	23718	26271	6198	8884	4890	7215
Adaptor Tag	1	83	1	28	1	22
Tag CopyNum = 1	516291	516291	233886	233886	226351	226351
Clean Tag	249407	7224604	126648	4272537	133936	5268280
CopyNum > 1	249407	7224604	126648	4272537	133936	5268280
CopyNum > 5	80567	6765151	48807	4057065	52715	5043703
CopyNum > 10	50762	6540885	32473	3933617	35585	4914261
CopyNum > 20	32580	6276679	21545	3774056	23869	4742946
CopyNum > 50	17820	5804733	11820	3459833	13697	4413595
CopyNum > 100	10562	5288358	6671	3092402	8166	4017505

### Analysis of tag mapping

**Figure 2:**
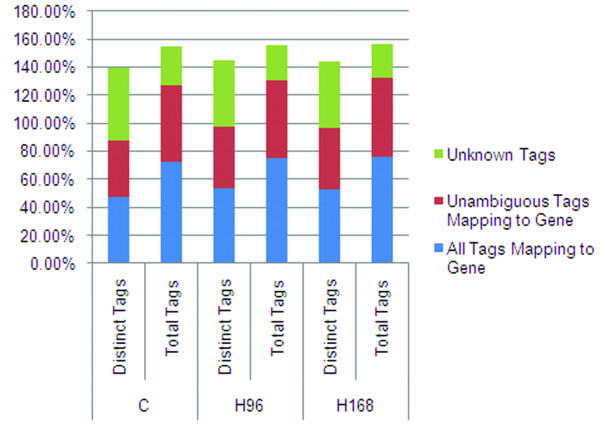
**Overview of tags mapping to the UniGene transcript database**. The vertical axis indicates the percentages of all and unambiguous tags mapped or not mapped to the UniGene virtual tag database. Unambiguous tags indicate the tags matched to only one gene.

For tag mapping, one reference tag database that included 51,670 sequences from *sus scrofa *Unigene was preprocessed. In order to obtain reference tags, NlaIII was used to digest the samples; the CATG + 17 tags in the gene were used as the gene's reference tags. We obtained 194,664 total reference tag sequences and 172,119 unambiguous tag sequences. Tolerances were set to allow one mismatch in each alignment to take into account polymorphism across samples. Using this approach, 47.71%~53.39% of distinct clean tags mapped to the Unigene virtual tag database, 39.42%~44.44% mapped unambiguously to the Unigene, and 52.29%~46.61% did not map to the Unigene virtual tag database (Figure 2). Incomplete pig genome sequencing is the most likely reason for the occurrence of unknown tags. Ideally, the Solexa experimental tags would be mapped to the CATG position closest to the 3' end, but for alternative splicing or incomplete enzyme digestion, these tags could map to a CATG position further along. Most of the Solexa experimental tags matched to the 1st or 2nd 3' CATG site in high-confidence transcripts (Figure S2 in Additional file 1). Depth analysis of transcriptome sampling in the DGE libraries demonstrated that the increased rate of all genes identified and genes identified by unambiguous tags declined significantly as the library increased in size. When the library size reached two million, 45% of all genes could be identified and 35% of genes were identified by unambiguous tags. At this time, library capacity approached saturation (Figure S3 in Additional file 1).

### Identification of differentially expressed (DE) genes and signaling pathway analysis

To identify global transcriptional changes in H-PRRSV infected porcine lungs, a previously described method [17] modified properly was utilized to identify DE genes from normalized DGE data via pairwise comparisons between differential time points (H96/C, H168/C, H168/H96) during infection: 4520 genes had p values < 0.005, the false discovery rate (FDR) was < 0.01 and the estimated absolute log2-fold change was > 0.5 in at least one of the pairwise comparisons, which were declared to be differentially expressed during the course of infection (Additional file 2).

**Figure 3:**
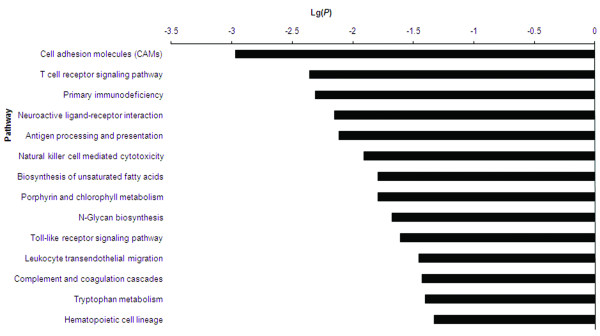
**Signaling pathways of DE genes**. Pathway analysis was predominantly based on the KEGG database. A P-value of < 0.05 and an FDR of < 0.05 in the two-sided Fisher's exact test were classed as significant. The vertical axis represents the pathway category and the horizontal axis represents the log10 (*p *value) of these significant pathways.

Pathway analysis of DE genes was performed using the Kyoto Encyclopedia of Genes and Genomes (KEGG) database and the two-sided Fisher's exact test. Only significant pathway categories that had a P-value of < 0.05 and an FDR of < 0.05 were analyzed. The significant signaling pathways included cell adhesion molecules (CAMs), T cell receptor signaling pathway, antigen processing and presentation, natural killer cell mediated cytotoxicity, Toll-like receptor signaling pathway, and complement and coagulation cascades (Figure 3; Additional file 3).

### Validation of DGE data using qPCR and serum cytokine analysis

**Figure 4:**
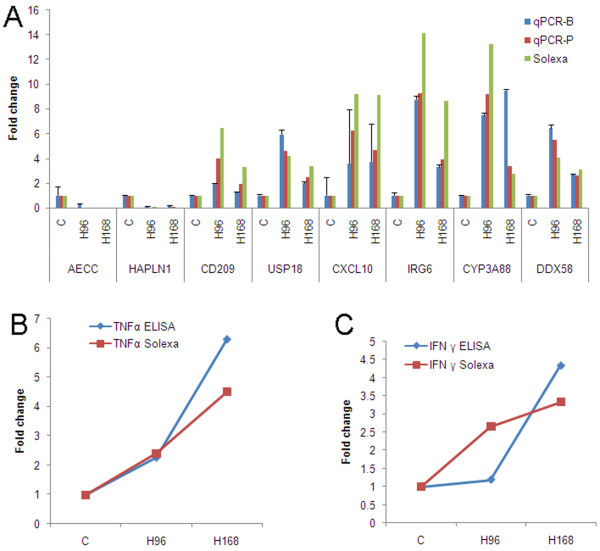
**Validation of DGE data by qPCR and serum cytokine analysis**. (A) qPCR validation of DGE data. Relative quantification was carried out to measure changes in target gene expression in lung samples relative to an endogenous reference sample. Results are expressed as the target/reference ratio of each sample normalized by the target/reference ratio of the calibrator; HPRT1 was used as a reference gene. The Y axis indicates the fold change of transcript abundance in H-PRRSV infected pigs compared to the C. For the C sample, the fold change of transcript abundance relative to the C sample equals one. qPCR-B: the RNA samples from independent extractions from biological replicates; qPCR-P: the RNA samples from pooled samples that were used for deep sequencing. Error bars represent SE. (B-C) the correlation between change in protein concentration from serum and transcript abundance from lungs. The levels of cytokines (TNFα and IFNγ) in serum were analyzed using ELISA. The levels at 96 and 168 hours were compared with the C to determine relative changes. The Y axis represents the relative fold change.

To validate DE genes identified by Solexa sequencing, eight genes were selected for confirmation using qPCR. The set included two down-regulated genes (epithelial chloride channel protein (AECC) and hyaluronan and proteoglycan link protein 1 (HAPLN1)) and six up-regulated genes (inflammatory response protein 6 (IRG6), DEAD (Asp-Glu-Ala-Asp) box polypeptide 58 (DDX58), ubiquitin specific peptidase 18 (USP18), chemokine C-X-C motif ligand 10 (CXCL10), cytochrome P450 (CYP3A88) and CD209). Data were presented as fold changes in gene expression normalized to the hypoxanthine phosphoribosyltransferase 1 (HPRT1) gene and relative to the C sample. Pearson's correlation coefficient (r) demonstrated that the DGE and qPCR data (pooling samples) were highly correlated; genes modulated by H-PRRSV had a very high consistency and r values ranged from 0.935 (USP18) to 1.000 (AECC) between the two methods (Figure 4A). qPCR analysis (pooling samples and independent RNA extractions from biological replicates) confirmed the direction of change detected by DGE analysis.

TNFα expression was elevated 2.27- to 6.29-fold in the sera of H-PRRSV-infected pigs on days 4 (p = 0.000) and 7 (p = 0.000) post infection, respectively, compared with C levels (Figure 4B). In H-PRRSV-infected animals, IFN-γ expression increased 1.2-fold by 3 d pi (p = 0.216), and 4.3-fold by 7 d pi (p = 0.000) (Figure 4C).

### STC (Series Test of Cluster) and STC-GO (Series Test Cluster of Gene Ontology) analysis

In order to profile the gene expression time series and search for the most probable set of clusters generating the observed time series, the STC algorithm of gene expression dynamics was used, which took into account the dynamic nature of temporal gene expression profiles during clustering and identified the number of distinct clusters. DE genes exhibited eight types of temporal expression pattern (Figure S4 in Additional file 1; Additional file 2) with four significant cluster profiles (profile 6,0,1,7), which have significantly more genes assigned under the true ordering of time points than the average number assigned to the model profile in the permutation runs (Figure S4 in Additional file 1). One striking observation was the relative constancy in gene expression profiles of four (6, 0, 1, 7) significant cluster profiles, particularly profiles 6 and 1. The sustained host response in H-PRRSV-infected pigs indicated that critical decisions influencing the outcome of infection occur very early after infection. Gene Ontology (GO) based on biological process (BP) enrichment analyses for sets of DE genes having significant cluster profiles was performed using the two-sided Fisher's exact test (Additional file 4; Additional file 5; Figure S5 and S6 in Additional file 1). Significant GO categories that had a P-value of < 0.05 were used.

The most prominently over-represented GO terms of significant cluster profile 6 (0,1,1, those are up-regulated genes) included pathogenesis, release of cytochrome c from mitochondria, complement activation, regulation of ubiquitin-protein ligase activity, mitochondrial transport, inflammatory response, innate immune response, defense response, response to stress, oxidation reduction, proteolysis, lipid metabolic process, cell communication, signal transduction, endocytosis, apoptosis, regulation of caspase activity, and regulation of interferon biosynthesis and production (Additional file 4). These results are consistent with these genes and their associated processes having important roles in H-PRRSV replication and pathogenesis.

The most prominently over-represented GO terms of significant cluster profile 1 (0,-1,-1, those are down-regulated genes) included epithelial cell differentiation, sterol, steroid, cholesterol, lipid biosynthetic and metabolic process, actin cytoskeleton reorganization, regulation of transport, cell proliferation and adhesion, and cellular biosynthetic process (Additional file 4). These results suggest that H-PRRSV infection could inhibit epithelial cell differentiation. Impaired regulation associated with the biosynthesis and metabolism of steroids, cholesterol and lipids indicated that they could be involved in H-PRRSV pathogenesis.

### Innate immunity

**Figure 5:**
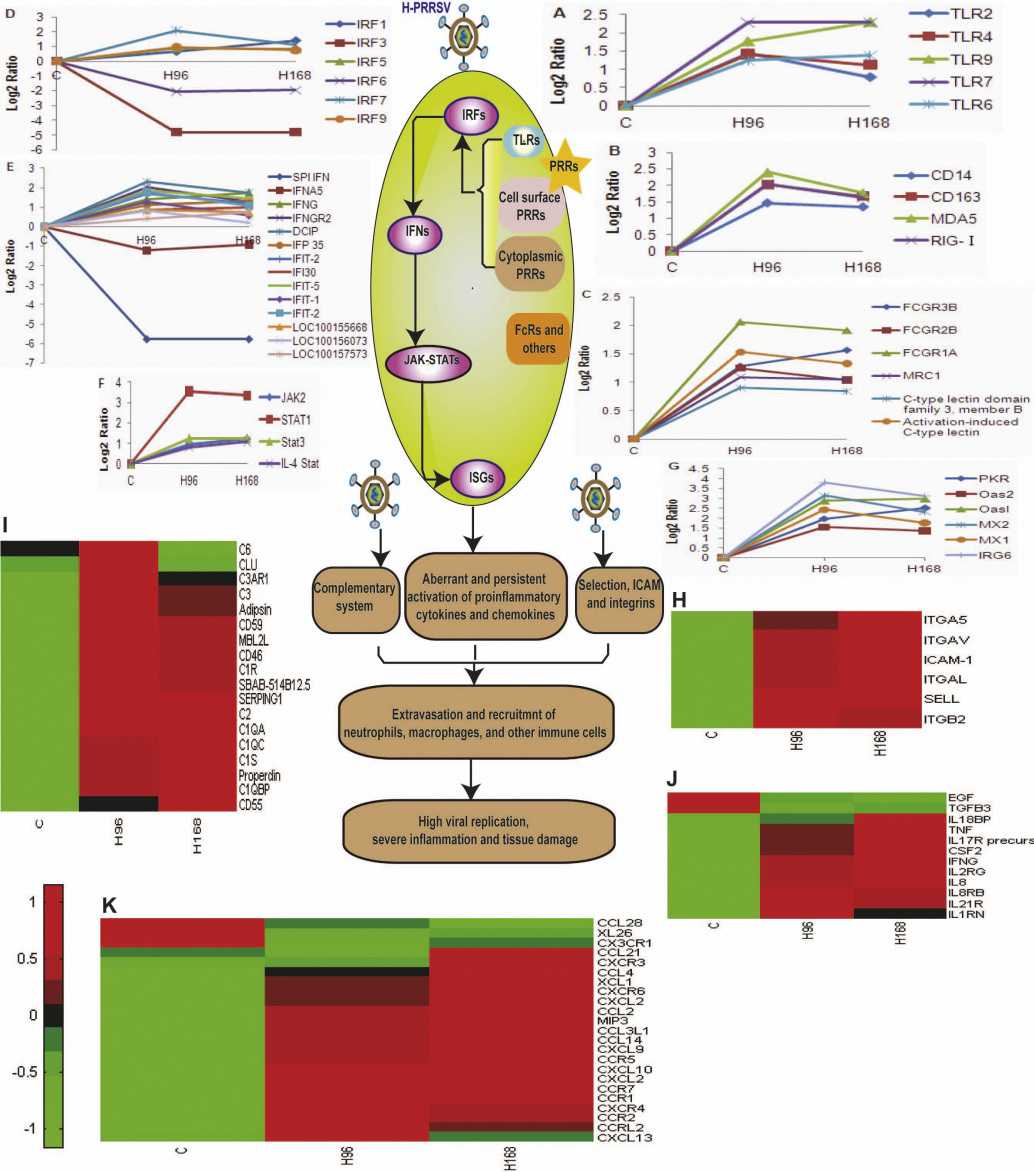
**Induction of innate immune responses in pigs infected with H-PRRSV**. PAMPs in H-PRRSV viral proteins and nucleic acids are recognized by PRRs that include TLRs (A), cell surface and cytoplasmic PRRs (B), Fc and other receptors (C). After binding to H-PRRSV viral PAMPs, PRRs initiate intracellular signaling cascades that activate transcription factors including IRFs (D) and STATs (F), which in turn induce the expression of IFNγ but not SPIIFNs (E), ISGs (G), pro-inflammatory cytokines and receptors (J), chemokines and receptors (K). Simultaneously, H-PRRSV infection activated complement proteins (I), selectin ligands, adhesion molecules and integrins (H). These recruit immune cells to sites of infection, resulting in severe inflammation and tissue damage. Figure 1H-K represent the heat maps of those DE genes selected. Genes shown in red were up-regulated and those shown in green were down-regulated in infected pigs relative to C pigs (See Additional file 2 for full gene names).

The antiviral response is triggered when host pathogen-recognition receptors (PRRs) are engaged by pathogen-associated molecular patterns (PAMPs) in viral proteins and nucleic acids [18]. Transcriptome analysis suggests that apparent reactive changes after H-PRRSV infection include activation of complement pathways, PRRs and other receptors potentially responsible for H-PRRSV recognition and uptake. As demonstrated in Figure 5, transcripts of the Toll-like PRRs TLR2, TLR4, TLR6, TLR7, TLR9 and TLR adaptor molecule 1 were significantly induced in H-PRRSV-infected pigs 4-7 d pi; no change was detected in expression of TLR3, which specializes in the recognition of viral dsRNA. Cytoplasmic PRRs DDX58 (retinoic-acid-inducible protein I, RIG-I) and melanoma differentiation-associated gene 5 (MDA5), the two most important PRRs for defense against viruses, were expressed at high levels after H-PRRSV infection. Cell surface PRRs such as CD14 and CD163 (thought to be involved in PRRSV entry during uncoating [19]) were likewise up-regulated after H-PRRSV infection. Moreover, three categories of Fc receptors (FCGR3B, FCGR2B, FCGR1A), mannose receptor C1 (MRC1) and c-type lectin were significantly induced in H-PRRSV-infected lungs. After binding to H-PRRSV viral PAMPs, PRRs initiated intracellular signaling cascades that activate transcription factors including IRF1, IRF5, IRF7, IRF9, and signal transducer and activator of transcription (STAT1, STAT3, STAT6) and JAK2 kinases; IRF3 was not activated. These transcription factors induced the expression of IFN-γ, IFN-stimulated genes (ISGs) including protein kinase R (PKR), 2',5'-oligoadenylate synthetase (OAS) and MX, and pro-inflammatory cytokines and chemokines; SPIIFNs were not induced. It is perplexing and paradoxical that H-PRRSV infection significantly induced expression of ISGs including IRF3 target genes, yet blocked the activation of IRF3 and SPIIFNs. Interestingly, induction of ISGs expression and blocking the activation of IRF3 has been observed in liver tissue from HCV-infected patients [18,20]. This poses an interesting question concerning the source of ISG expression, suggesting that ISGs are predominantly expressed in uninfected cells. T cells and plasmacytoid dendritic cells that infiltrate the liver are a possible source of hepatic type IIFNs [20]. Simultaneously, H-PRRSV infection activated complement proteins (C1, C2, C3, C4, C6, and mannose-binding lectin 2-like isoform2) that could contribute to the production of channels in a lipid bilayer and result in the lysis of viruses, and up-regulated mRNA expression of regulatory proteins of complement activation [21] (CD46, CD55, and CD59), which could increase the resistance of viral serum and benefit progeny virus. Complement complexes and PRRs could be responsible for stimulating the production of inflammatory cytokines and chemokines that recruit neutrophils, macrophages and other immune cells to sites of infection; selectin ligands, adhesion molecules and integrins participate in the process. Expression of genes including those for selectin L (SELL), intercellular adhesion molecule-1 (ICAM-1), integrin alpha L (ITGAL), ITGAV, ITGA5 and integrin beta-2 precursor (ITGB2) was up-regulated. These results suggest that extravasation and recruitment of immune cells is a multistep process that involves the increased expression of genes including those for CAMs, intracellular signaling molecules, cytokines and their receptors, chemokines and their receptors.

### Adaptive immunity

**Figure 6:**
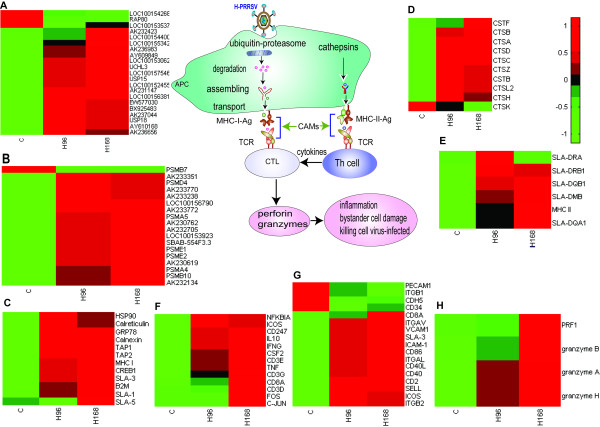
**Induction of adaptive immune responses in pigs infected with H-PRRSV**. (A) Ubiquitin-proteins and ubiquitin enzymes; (B) proteasome; (C) genes related to assembly and transport of MHC-I-Ag complex; (D) cathepsins; (E) genes involved in antigen presentation to MHC class IImolecules in H-PRRSV infection; (F) genes related to T cell receptor (TCR) signaling pathway; (G) cell adhesion molecules (CAMs); (H) perforin (PRF1) and granzymes. Genes shown in red were up-regulated and those shown in green were down-regulated in infected pigs relative to C pigs (See Additional file 2 for full gene names).

T cells only recognize antigens that are bound to major histocompatibility complex (MHC) proteins on the surface of other cells. Therefore, responses to foreign protein antigens begin after the antigen has been captured, processed and presented by these cells. The scope of MHC gene activation in H-PRRSV infection was investigated by analyzing the expression of genes that process and present antigens via the class I or class II pathway. Antigens from intracellular pathogens that bind MHC class I molecules are subjected to a sequence of events that include ubiquitination, degradation and transportation. Various ubiquitin-proteins (including ubiquitin specific peptidase 18 (USP18) and USP15) and ubiquitin enzymes (such as ubiquitin-conjugating enzymes E2 and E3) were significantly up-regulated in H-PRRSV infected lungs (Figure 6). Seventeen of the 18 DE proteasomes, a complex cytoplasmic organelle that breaks down proteins into peptides and delivers them across internal membranes into the endoplasmic reticulum, were significantly up-regulated in H-PRRSV infected lungs. Loading of small peptide fragments on to MHC class I molecules, which were significantly and coordinately up-regulated (Figure 6), required efficient assembly and transport by chaperone molecules such as beta-2-microglobulin (B2M), transporter associated with antigen processing 1 (TAP1), TAP2, calnexin, calreticulin, glucose-regulated protein 78 kDa (grp78) and 90-kDa heat shock protein (HSP90). Similarly, class II antigen-processing genes including cathepsins and genes involved in class II presentation such as SLA-DQ, SLA-DR and SLA-DM were highly expressed in H-PRRSV infected lungs, relative to C.

Cytotoxic T-lymphocytes (CTLs) are the major cell-mediated immune response to viral infections and are MHC restricted. Clones of CTL cells recognize a specific antigen when it is presented to the T-cell receptor (TCR)/CD3 complex on the surface of the CTL by MHC I on the surface of the target cell. CT activity requires 'help' (i.e. cytokine production) from T-helper (Th) lymphocytes. TCRs of Th lymphocytes recognize specific antigens presented by MHC II molecules on antigen presenting cells (APCs). T cell activation requires TCR signals and co-stimulators. Co-receptor molecules and CAMs ensure that APCs are in contact with T cells for a substantial time, enhancing the interactions of APCs and T cells. Gene expression of TCR signals (CD3, IFNG, TNF, NF-kappa-B inhibitor alpha (NFKBIA) and IL10) and co-stimulators (inducible T-cell co-stimulator (ICOS) and CD86) were significantly up-regulated in H-PRRSV infected lungs (Figure 6). Furthermore, gene expression of co-receptor molecules (CD8A) and CAMs (CAM-1, VCAM1, CD40, CD2, ITGAL, ITGAV, ITGB2 and SELL) increased significantly. Collaborative action of TCR signals, co-stimulators, co-receptor molecules and CAMs leads to activation of Th cells. Activated Th cells produced cytokines (IFN-γ, TNF and IL10) and expressed CD40L, which bound to CD40 on APCs to activate them; activated APCs are more efficient in stimulating the differentiation of CD8+ T cells. Through recognition of peptide-class I MHC complexes by the TCR and involvement of the CD8 co-receptor, co-stimulator molecules and Th cells, naïve CD8+ T cells differentiated into functional CTLs capable of recognizing and 'killing' target cells bearing the same epitope on their MHC class Imolecules. Activated CTLs release perforin (PFR1) and granzymes to kill target cells. Gene expression for PRF1 and granzymes B, A and H were significantly up-regulated in H-PRRSV infected lungs, relative to C (Figure 6).

### Cell death

**Figure 7:**
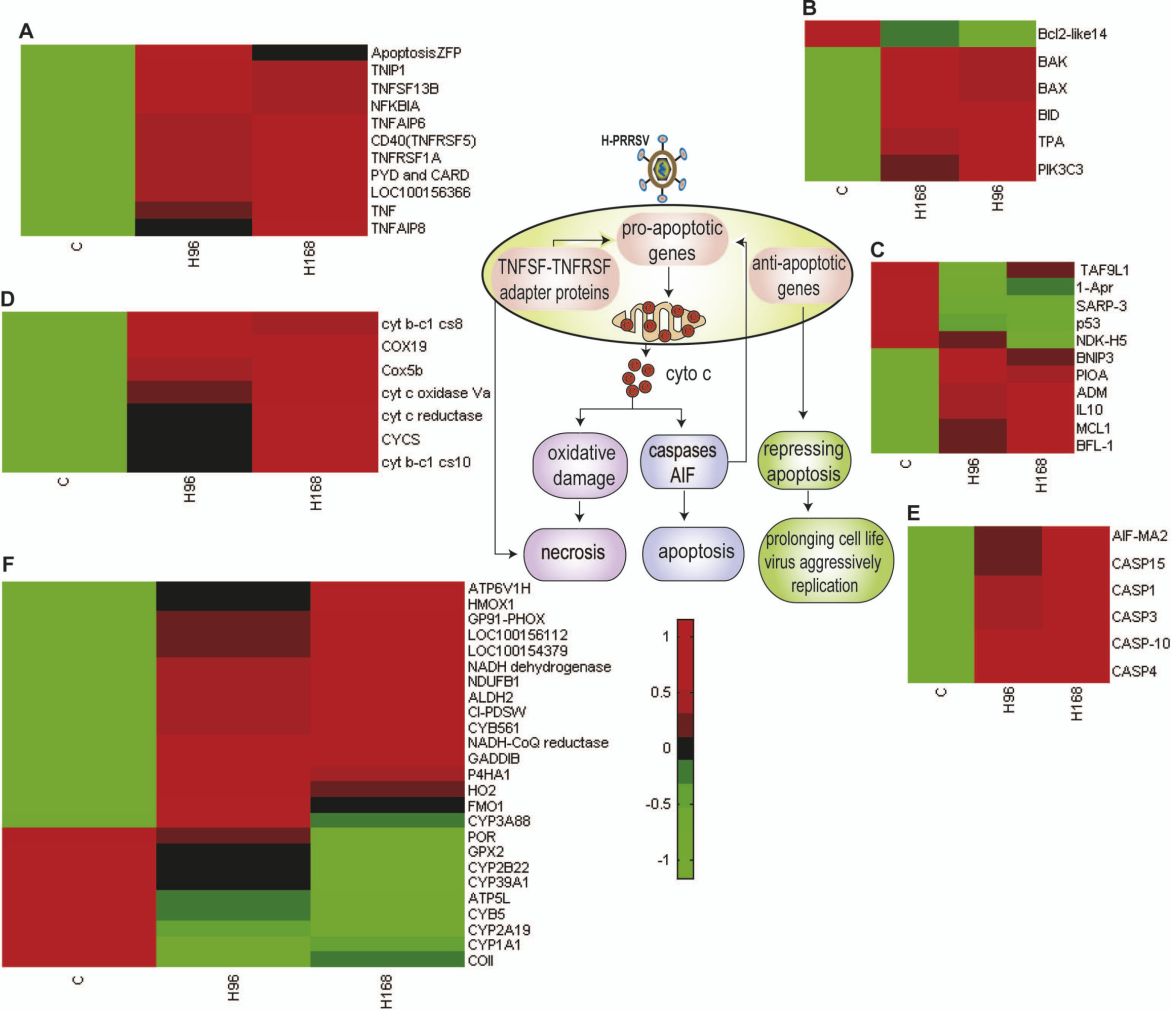
**Schematic of cell death induced by H-PRRSV infection**. The heat maps represent expression of selected DE genes associated with (A) tumor necrosis factor TNFSF, TNFRSF and adaptor proteins, (B) pro-apoptotic, (C) anti-apoptotic, (D) cytochrome c, (E) caspases and apoptosis-inducing factor (AIF), (F) electron transport and oxidative damage. Genes shown in red were up-regulated and those shown in green were down-regulated in infected pigs relative to C pigs (See Additional file 2 for full gene names).

Apoptosis is considered to be an important host defense mechanism that interrupts viral replication and eliminates virus-infected cells. Viruses often kill infected cells by inducing apoptosis rather than necrosis, but some viruses can repress apoptosis to prolong the life of the cell and increase the yield of progeny virions. H-PRRSV infection up-regulated expression of the TNF superfamily (TNFSF), TNF receptor superfamily (TNFRSF) and adapter proteins including TNF, TNFR1, NFKBIA, PYD and CARD domain containing apoptosis response zinc finger protein, which directly result in cell death (Figure 7). H-PRRSV infection caused up-regulation of pro-apoptotic proteins including BAX, BAK, BID and 3 phosphoinositide-3-kinase (PIK3C3). Up-regulation of pro-apoptotic proteins could result in disruption of the mitochondria transmembrane potential, thereby inducing release of cytochrome c, apoptosis-inducing factor (AIF)-like mitochondrion-associated inducer of death (LOC100153541), caspase-10 precursor (CASP-10), CASP1, CASP4, CASP15 and CASP3 from mitochondrial membranes, leading to the induction of apoptosis and secondary necrosis. Mitochondria are the major producers of reactive oxygen species, particularly superoxide radicals, which cause oxidative damage to cells and tissues. In H-PRRSV infected lungs the expression of genes involved in significant oxidative damage such as cytochrome b245 heavy chain (p22 phagocyte B-cytochrome GP91-PHOX), hemoxygenase-1 (HMOX1), growth arrest and DNA-damage-inducible beta isoform CRA_b (GADDIB) were up-regulated; gene expression of the antioxidant glutathione peroxidase 2 (GPX2) was significantly down-regulated.

H-PRRSV induced up-regulation of anti-apoptotic genes in infected lungs including the BCL2-related myeloid cell leukemia sequence 1 (MCL1), Bcl-2-related protein A1 (protein BFL-1), putative inhibitor of apoptosis, adrenomedullin (ADM) and IL10, and the down-regulation of pro-apoptotic genes including p53 protein, apoptosis-inducing TAF9-like domain 1, apoptosis-related protein 1 (APR-1), secreted apoptosis-related protein 3 (SARP-3) and nucleoside diphosphate kinase homolog 5 (NDK-H 5). These actions of H-PRRSV serve to inhibit apoptosis, possibly prolonging the life-span of the cell and thereby increasing the yield of progeny virions.

## Discussion

The results from the present study are in agreement with previous research that demonstrated that H-PRRSV infected pigs exhibit severe clinical symptoms including persistent high fever, reddening of the skin, conjunctivitis, dyspnoea and severe diffuse pulmonary consolidation lesions [2,22]. Histopathological examination demonstrated robust interstitial pneumonia in the lungs with thickening of alveolar septa accompanied by extensive infiltration of immune cells (Figure 1). The H-PRRSV virus replicates prolifically in the lungs, spleen and lymphoid organs. During infection an invading virus is recognized by PRRs that engage PAMPs and trigger signaling pathways within infected cells that are involved in innate immune (Figure 5) and adaptive immune (Figure 6) responses. Host immune responses are normally protective but if numerous cells are infected before immune induction, immune-mediated destruction can result in severe or fatal pathological consequences. Global profiling of transcriptional changes occurring in host lungs during H-PRRSV viral infection, analyzed using high-throughput Solexa sequencing, has provided important information regarding how H-PRRSV viruses trigger and regulate host immune responses and cause disease.

QPCR assays demonstrated that the H-PRRSV virus replicated rapidly and persisted in infected cells (Table S1 in Additional file 1). Substantial viral antigen was detected in alveolar cells and bronchiolar epithelial cells (Figure 1). The ability of a virus to induce and sustain an infection depends partly on its ability to block host innate immune responses or to modulate the activity of antiviral effector proteins. Production of type I IFN (IFN-α/β, SPI IFN) is an innate antiviral immune reaction in virus-infected cells that prevents viral replication and restricts the spread of the virus to neighboring cells. However, the present study demonstrated that H-PRRSV infection suppressed production of SPI IFN and down-regulated expression of IFN-α (Figure 5E). Previous *in vitro *and *in vivo *studies [8,23,24] have demonstrated that PRRSV infection results in minimal IFN-α production or suppresses its production, and IFN-α has been shown to inhibit PRRSV replication. During H-RRRSV viral infection, blocking SPI IFN production and particularly production of IFN-α could result in rapid spread of the virus and a high rate of viral replication. Other viral infections including the 1918 influenza virus [25], hepatitis C virus (HCV) [20] and Ebola virus [26] suppress type I IFN gene expression, leading to extensive viral replication and increased pathogenesis. IRF3 plays an important role in typeI IFN gene expression and the present study demonstrated that IRF3 gene expression was suppressed during H-PRRSV infection (Figure 5D). This result is in agreement with a previous study reporting that PRRSV NSP1β inhibited IRF3 and NF-κB transactivation, and down-regulated IFN-β gene expression[27]. This suggested that NSP1β mediates subversion of the host innate immune response and plays an important role in PRRSV pathogenesis. Furthermore, influenza A NSP1 can suppress innate immunity by inhibiting activation of IRF3, and subsequently disrupting the induction of α/β-interferon [28].

Many viruses induce apoptosis in infected cells but some can block the apoptosis pathway, leading to prolonged life of the cell and an increase in the yield of progeny virions. H-PRRSV up-regulated expression of anti-apoptotic genes and down-regulated expression of some pro-apoptotic genes in H-PRRSV infected lungs (Figure 7C). MCL1, BFL-1, putative inhibitor of apoptosis, ADM and IL10 were up-regulated. MCL1 and BFL-1 belong to the BCL-2 subfamily, which negatively regulates apoptosis and blocks the apoptosis pathway; ADM is an anti-apoptotic peptide [29]; and IL10 protects cells against apoptosis [30]. The pro-apoptotic genes APR-1, p53 protein, SARP-3, and NDK-H 5 were down-regulated to prevent the occurrence of apoptosis. These findings indicate that H-PRRSV could induce an anti-apoptotic state to prolong the life-span of infected cells and increase the yield of progeny virions.

IL10 could have an important role in the regulation of the immune response to PRRSV. Up-regulation of IL10 gene expression has been demonstrated in PRRSV-infected porcine leukocytes, alveolar macrophages, dendritic cells, and *in vivo *in PRRSV infected pigs [8,31]. Incubation of freshly isolated CD14 positive cells with IL10 during differentiation increased susceptibility to PRRSV infection and was correlated with up-regulation of CD163 on the cell surface [32]. This suggests that IL10 plays an important role in CD163 up-regulation and susceptibility to PRRSV during differentiation of macrophages *in vivo*. CD163 alone can confer PRRSV replication on a non-permissive pig cell line and its expression on macrophages *in vivo *could determine the efficiency of replication and subsequent pathogenicity of PRRSV [32]. It is possible that internalization of H-PRRSV via CD163 on the target cells could induce expression of IL10 and subsequently induce the expression of CD163 on neighboring undifferentiated monocytes, increasing overall susceptibility to PRRSV.

Taken together, the above findings suggested that the H-PRRSV virus aggressively replicated and disseminated by subverting the host innate immune response, inducing an anti-apoptotic state and up-regulating expression of CD163.

Prolific replication and rapid spread of H-PRRSV virus caused severe lung damage, hemorrhage and extensive infiltration of immune cells throughout the course of infection. Accordingly, significant increases in the expression of a number of genes involved in phagocytic cell activation were observed including CAMs, and several pro-inflammatory cytokines and chemokines such as IFN-γ, TNF, SELL, ICAM, integrin, C-type lectin, IL2RG, IL8, CSF2, IRG6, macrophage inflammatory protein 3 (MIP-3), CXCL2, CXCL9, CXCL10, CCL2 and CCR5 (Figure 5H, J, K). Up-regulated expression of these genes resulted in recruitment of neutrophils, macrophages and other immune cells to sites of infection, and excessive infiltration resulted in destruction of tissues [33]. Moreover, H-PRRSV infection resulted in the activation of CD4 and CD8 T lymphocytes specific for H-PRRSV antigens, and these secreted vasoactive cytokines including TNFα and IFN-γ. This cytokine 'storm' increased capillary fragility (with associated hemorrhages) and permeability. H-PRRSV infection activated complement proteins, which enhanced vascular permeability and were associated with sequestration of thrombocytes. The sustained induction of pro-inflammatory cytokines and chemokines contributed to a robust inflammatory response in the lung.

Fever is frequently the initial response to infection and it is triggered by PRR-PAMP interactions that activate a signaling cascade that causes the production of inflammatory cytokines responsible for fever including CASP1, the IL1-converting enzyme responsible for cleaving the IL-1β precursor and resulting in production of the mature form [34]. TLR2, 4, 6, 7, 9 (Figure 5A) and CASP1 (Figure 7E) were significantly up-regulated in H-PRRSV infected lungs. Heat shock proteins, referred to as stress proteins, are induced in cells exposed to a wide range of environmental stressors including infection and extreme temperature. Gene expression levels of heat shock genes including HSPA5, HSP27, HSP90, HSP90B1, HSPCB and HSPD1 were significantly elevated in H-PRRSV infected lungs relative to C (Figure S7 in Additional file 1).

During H-RRRSV virus infection, activated CTLs and NK cells release perforin and granzymes to kill target cells. Gene expression of PRF1 and granzyme B, A and H were significantly up-regulated in H-PRRSV infected lungs (Figure 6H). Perforin is exocytosed and polymerizes in the target cell plasma membrane to form pores. Granzymes enter target cells through the perforin pores and induce target cell apoptosis. The perforin pores also allow the release of intracellular calcium from the target cell, which acts to trigger apoptotic pathways. The induction of a CTL response results in the release of various cytokines from Th cells, some of which result in clonal proliferation of antigen-specific CTLs, and others that have direct antiviral effects. Diffusion of perforin and local cytokine production frequently results in inflammation and bystander cell damage.

There was up-regulation of genes involved in TNF signaling including TNFα and TNFRSF1A (Figure 7A) and the up-regulation of TNF during PRRSV infection has been reported to have an important role in pathogenicity. It has been reported that PRRSV infection is a potent inducer of TNFα in PAMs [35]. In the present study, continuously up-regulated expression of TNFα (at mRNA and protein levels) from 96 h pi to 168 h pi was observed (Figure 4B). Interestingly, infection with H-PRRSV led to up-regulation of NFKBIA, an inhibitor of the TNF receptor activated transcription factor NF-κB. Loss of NF-κB activity has been reported to increase the cytotoxic effects of TNF and result in increased cell death [36]. TNF and NFKBIA could act synergistically to cause significant alveolar and bronchial epithelial cell necrosis during H-PRRSV infections.

This study has indicated that H-PRRSV could induce apoptosis through a mitochondria-mediated pathway, and previous research provided evidence that PRRSV induces apoptosis in MARC-145 cells through an intrinsic mitochondria-mediated pathway [37]. Pro-apoptotic genes (BAX, BAK, BID, PIK3C3), cytochrome C, and caspases (CASP-10, CASP1, CASP4, CASP15, CASP3) were up-regulated (Figure 7). These results indicate that up-regulation of pro-apoptotic genes resulted in disruption of the mitochondrial transmembrane potential, thereby inducing release of cytochrome c, AIF-like mitochondrion-associated inducer of death and CASP3 from mitochondrial membranes, leading to induction of apoptosis and secondary necrosis. The release of cytochrome c can also induce necrosis through a slower non-apoptotic mechanism due to the electrochemical gradient across the inner membrane, production of reactive oxygen species (ROS) and declining ATP production [38]. The production of ROS, particularly superoxide radicals, and the subsequent oxidative damage to cells and tissues, are recognized as key contributors to viral pathogenesis [36,39]. ROS-mediated oxidative stress could also contribute to PRRSV-induced apoptosis [37]. In the current study, continuous up-regulated expression of cytochrome b245 heavy chain (GP91-PHOX), a critical component of the membrane-bound oxidase of phagocytes (macrophages and neutrophils) that generates superoxide radicals, was observed from 96 h pi to 168 h pi (Figure 7F). Increased expression of cytochrome b245 in H-PRRSV infected lungs implies the increased production of oxygen radicals and the activation of phagocytic cells. Taken together, these data suggested that the severe pulmonary pathology caused by H-PRRSV infection was induced by significant production of TNF, PRF1, granzymes, cytochrome c and oxygen radicals.

## Conclusions

**Figure 8:**
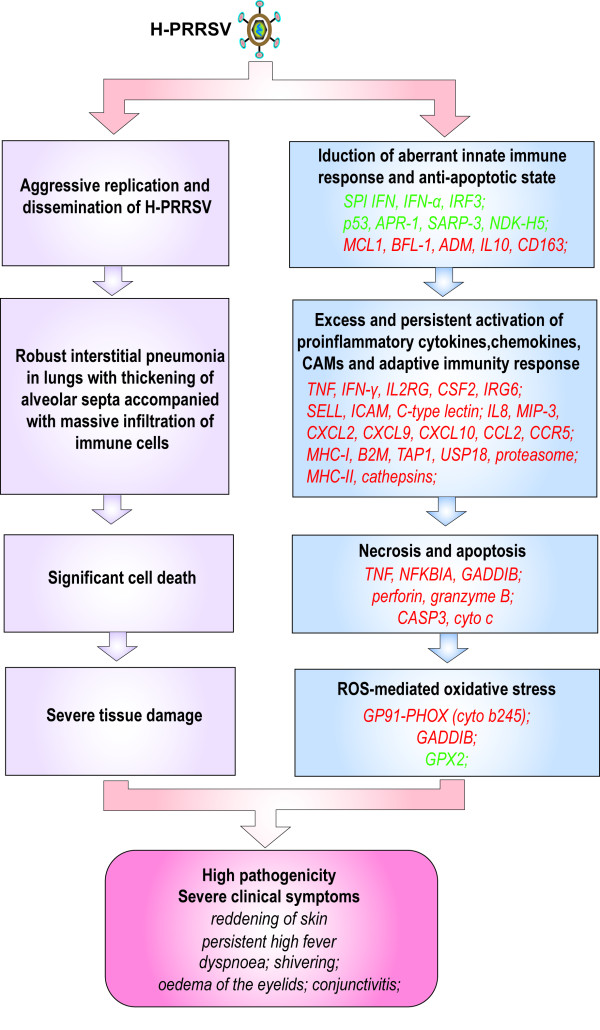
**Model of the relationship between pulmonary gene expression profiles and infection pathology**. Genes shown in red were up-regulated and those shown in green were down-regulated in infected pigs relative to C pigs (See Additional file 2 for full gene names).

From the data presented herein, a model summarizing the relationship between pulmonary gene expression profiles and infection pathology has been proposed (Figure 8). H-PRRSV virus replicated prolifically and disseminated by inducing an aberrant innate immune response and an anti-apoptotic state, as evidenced by suppressed expression of SPI IFN, IFN-α, IRF3, and pro-apoptotic genes including p53, APR-1, SARP-3 and NDK-H5. Furthermore, expression of anti-apoptotic genes for MCL1, BFL-1, ADM, IL10 and CD163 was up-regulated. Prolific replication and rapid spread of H-PRRSV virus resulted in a vigorous inflammatory response as indicated by aberrantly high and sustained expression of proinflammatory cytokines and chemokines, CAMs and genes associated with adaptive immune response including TNFα, IFN-γ, IL2RG, IL8, CSF2, IRG6, SELL, ICAM, C-type lectin, MIP-3, CXCL2, CXCL9, CXCL10, CCL2, CCR5, MHC-I, B2M, TAP1 and MHC- II. This was compounded by significant cell death and elevated expression of TNF, NFKBIA, GADDIB, perforin, granzyme B, CASP3 and cytochrome c, coupled with increased ROS-mediated oxidative stress as indicated by up-regulation of cytochrome b245 and HMOX1, and down-regulation of the antioxidant GPX2. H-PRRSV replicated rapidly resulting in excessively vigorous immune and inflammatory responses that contributed to severe tissue damage, high pathogenicity and in some cases, death. The systems analysis carried out here provides a comprehensive basis for a better understanding of the pathogenesis of H-PRRSV and the identification of genetic components involved in H-PRRSV resistance/susceptibility in swine populations.

## Methods

### Experimental animals and tissue collection

All animal procedures were performed according to guidelines developed by the China Council on Animal Care and protocols were approved by the Animal Care and Use Committee of Guangdong Province, China. Nine conventionally-reared healthy 6-week-old crossbred weaned pigs (Landrace × Yorkshire) were selected from a high-health commercial farm that has historically been free from all major pig diseases including PRRSV, porcine circovirus type 2, classical swine fever virus, porcine parvovirus, pseudorabies virus, swine influenza virus and *Mycoplasma hyopneumoniae *infections. All pigs were PRRSV-seronegative as determined by ELISA (HerdChek PRRS 2XR; IDEXX Laboratories) and absence of PRRSV was confirmed by RT-PCR. Pigs were randomly assigned to three groups and raised in isolation rooms. Six pigs were inoculated with 6 ml viral suspension (4 ml intranasally and 2 ml intramuscularly) of H-PRRSV (H-PRRSV gift from Dr. Zhang Guihong, South China Agricultural University) at a dose of 10^6.0 ^TCID50 ml^-1 ^on day 0. Three C pigs were treated with an identical volume of DMEM culture medium from uninfected MARC-145 cells 1 day prior to experimental infection, and were immediately necropsied. H-PRRSV-inoculated pigs were clinically examined daily and rectal body temperatures were recorded from day 2 to 7 pi. Viral re-isolates and H-PRRSV specific RT-PCR were performed after the pigs were sacrificed. Three infected pigs randomly chosen were necropsied at 96 h pi and 168 h pi. Lung samples were collected from C, H96 and H168, and frozen in liquid nitrogen for RNA isolation or fixed in 10% neutralized buffered formalin for histological processing.

### Virus re-isolation and QPCR detection

Heart, liver, spleen, lung, kidney, lymph and brain tissue were collected at autopsy. Samples were placed in sterilized PBS buffer, homogenized and centrifuged to harvest supernatants for virus re-isolation and detection of H-PRRSV using QPCR. Tissue homogenates were inoculated on monolayers of MARC-145 cells and maintained in Dulbecco's modified Eagle's medium (DMEM) supplemented with 75 μg of penicillin and 50 μg of streptomycin per ml. Cytopathic effects (CPE) were monitored daily. Cultures not displaying CPE after three passages were considered negative. Tissue homogenates were examined by H-PRRSV-specific QPCR. The oligonucleotide primers used were NSP2F2 (5'-GTGGGTCGGCACCAGTT-3') and NSP2R2 (5'- GACGCAGACAAATCCAGAGG-3'), designed from the gene segment encoding for NSP2 with a deletion of 87 bases in the fixed site as compared to the NA PRRSV. The TaqMan probe, 5'FAM-CGCGTAGAACTGTGACAACAACGCTGA-TAMRA3', was synthesized according to previous protocols [40].

### Histopathology

Lungs of C and experimentally infected pigs were processed for haematoxylin and eosin (H&E) and immunohistochemistry staining, as described previously [41].

### RNA extraction, library construction and sequencing

Total RNA was extracted from frozen lungs using standard protocols (Trizol) and treated with DNase to remove potential genomic DNA contamination, according to the manufacturer's protocol. RNA integrity and concentration were evaluated using an Agilent 2100 Bioanalyzer (Agilent Technologies, Palo Alto, CA, USA).

For RNA library construction and deep sequencing, RNA samples were prepared as follows: for each time point equal quantities of RNA isolated from three individual lungs were pooled from the H-PRRSV-inoculated group and the C group. A 6 μg sample of RNA from each group was submitted to Solexa (now Illumina Inc.) for sequencing.

Sequence tag preparation was carried out using Illumina's Digital Gene Expression Tag Profiling Kit according to the manufacturer's protocol. In brief, mRNA was isolated from 6 μg of total RNA by binding the mRNA to a magnetic oligo bead. First- and second-strand cDNA were synthesized while the mRNA was attached to the beads. Double stranded cDNA was digested with NlaIII to remove all fragments other than the 3' most CATG fragment attached to the oligobead. GEX NlaIII Adapter 1 was ligated at the site of NlaIII cleavage. GEX NlaIII Adapter 1 contains the sequence for the restriction enzyme MmeI, and the restriction enzyme MmeI was used to create the 17 bp tag. The GEX Adapter 2 was ligated at the site of MmeI cleavage. A 12 cycle PCR was performed with two primers that anneal to the ends of the adapters to enrich the adapter-ligated cDNA construct. The amplified cDNA construct was purified from a 6% Novex TBE PAGE gel. The purified cDNA tags were sequenced on the Illumina Cluster Station and Genome Analyzer. Image recognition and base calling were performed using the Illumina Pipeline.

### Analysis of sequencing data

For the raw data adaptor tags, low quality tags and tags of copy number = 1 were filtered to produce clean tags. The raw data (tag sequences and counts) have been submitted to Gene Expression Omnibus (GEO) under series GSE19456. The clean tags were classified according their copy number in the library and their percentages in the total clean tags were provided. Saturation of the library was also analyzed.

### Tag mapping

The pre-processed database of all possible CATG+17-nt tag sequences was created using *sus scrofa *UniGene (http://www.ncbi.nlm.nih.gov/UniGene/UGOrg.cgi?TAXID=9823, UniGene Build #35 *Sus scrofa*, Nov, 7th, 2008) from NCBI. Clean tags were aligned to the reference sequences, and unambiguous tags were annotated. The clean tag number corresponding to each gene was counted.

### Differential expression detection

To compare the differential expression of genes across samples (H96/C, H168/C, H168/H96), the number of raw clean tags in each sample was normalized to Tags Per Million (TPM) to obtain normalized gene expression levels. Differential expression detection of genes or tags across samples was performed [17]. Genes were classed as significantly differentially expressed if they had a P-value < 0.005, a false discovery rate (FDR) < 0.01 and an estimated absolute log2-fold change > 0.5 in sequence counts across libraries.

### qPCR and serum cytokine analysis

The RNA samples used for the qPCR assays were the same as those used for the DGE experiments and independent RNA extractions from biological replicates. qPCR was carried out on the Lightcycler480 (Roche) with SYBR-Green detection (SYBR PrimeScript RT-PCR Kit, TaKaRa Biotechnology Co., Ltd.), according to the manufacturer's instructions. Each cDNA was analyzed in triplicate, and the average threshold cycle was calculated. Relative expression levels were calculated using the 2^-ΔΔCt ^method. The results were normalized to the expression level of HPRT1 and relative to the C sample. Levels of cytokines (TNFα and IFN-γ) from serum were assayed using swine commercial ELISA kits from R&D Systems according to the manufacturer's instructions.

### STC and STC-GO analysis

STC is implemented entirely in java. The clustering algorithm selects a set of distinct and representative temporal expression profiles. These model profiles are selected independently of the data. The clustering algorithm assigns each gene passing the filtering criteria to the model profile that most closely matches the gene's expression profile as determined by the correlation coefficient. Since the model profiles are selected independently of the data, the algorithm can determine which profiles have a statistically significant higher number of genes assigned using a permutation test. This test determines an assignment of genes to model profiles using a large number of permutations of the time points. It uses standard hypothesis testing to determine which model profiles have significantly more genes assigned under the true ordering of time points compared to the average number assigned to the model profile in the permutation runs. Significant model profiles can be either analyzed independently or grouped together on the basis of similarity to form clusters of significant profiles [42,43].

STC-GO supports Gene Ontology enrichment analyses for sets of genes having the same significant temporal expression pattern. Random samples of *S*_*a *_(*S*_*a *_is the number of genes assigned to the same model temporal expression profile r) were selected and genes at each iteration and Fisher's exact test p-values for the selected genes in all GO biological categories were calculated [44]. The two-sided Fisher's exact test p-value for a category reflects a test of the null hypothesis that the category is enriched in genes assigned to profile r with respect to what would have been expected by chance alone. To decide whether to investigate a category that appears enriched in these genes further, the statistical reliability of the apparent enrichment would be calculated. To assess the significance of a particular category, the distribution of p-values that would occur by random chance must be known. The percentage of false positives to be tolerated will generally depend on the relative costs of false positives and false negatives in whatever follow-up study is to be carried out. This way of framing the question leads us to specify the false discovery rate (FDR) for a set of categories, rather than the significance level (p-value) for each category. With the significance at the 0.05 level for a given category, the enrichment R_e _is given by R_e _= (*i/m*)/(S_a_/*N*) where *i *is the number of genes assigned to profile r within the GO category of interest, m is the total number of genes within the GO category of interest, and N is total number of unique genes in the gene reference database list.

### Pathway analysis

Pathway analysis was predominantly based on the Kyoto Encyclopedia of Genes and Genomes (KEGG) database. The two-side Fisher's exact test with multiple testing and the χ^2 ^test were used to classify the pathway category. The false discovery rate (FDR) was used to correct the P-value. Only pathway categories that had a P < 0.05 were chosen. Within the significant category, the enrichment Re was given by: Re=nfnNfN (Re = ENRICHMENT), where *n*_*f *_is the number of flagged proteins within the particular category, n is the total number of proteins within the same category, *N*_*f *_is the number of flagged proteins in the protein reference database list, and N is the total number of proteins in the gene reference database list.

## Abbreviations

H-PRRSV: highly virulent porcine reproductive and respiratory syndrome virus; DGE: Digital Gene Expression tag profiling; DE: differentially expressed; QPCR: Quantitative PCR.

## Authors' contributions

XS designed the study, carried out the study and drafted the manuscript. MD participated in data analysis and revised the manuscript. WQ participated in data analysis and carried out qPCR. JJ participated in the design of the study and carried out the ELISA. QL participated in data analysis and revised the manuscript. YX participated in data analysis and revised the manuscript. NY participated in data analysis and revised the manuscript. ZX participated in data analysis and revised the manuscript. LX participated in data analysis and revised the manuscript. CY conceived the study and participated in its design and coordination. All authors read and approved the final manuscript.

## Supplementary Material

Additional file 1 **Seven supplementary figures and one supplementary table**. Additional file 1 contains seven supplementary figures and supplementary table 1 in PDF format. Figure S1. Saturation of DGE libraries. Figure S2. The positions of tags in the gene. Figure S3. Effect of library size on the number of genes identified. Figure S4. STC (Series Test of Cluster) analysis of DE genes. Figure S5. Biological process GO terms of profiles 6 and 1. Figure S6. Biological process GO terms of profiles 7 and 0. Figure S7. Differential expression of heat shock genes. Table S1. Tissue distribution of H-PRRSV in infected pigs using QPCR assays. Click here for file

Additional file 2 **Differentially expressed (DE) genes identified**. Additional file 2 contains 4520 DE genes identified via pairwise comparisons between differential time points (H96/C, H168/C, H168/H96) during the course of H-PRRSV infection in xls format. Click here for file

Additional file 3 **Pathway analysis of DE genes**. Additional file 3 contains details of pathway analysis of DE genes. The yellow color represents significant pathway categories that had a P-value of < 0.05 and an FDR of < 0.05. Click here for file

Additional file 4 **Series Test Cluster of Gene Ontology (STC-GO) analysis of profiles 6 and 1**. Additional file 4 contains details of GO based on biological process (BP) enrichment analyses for sets of DE genes of significant cluster profiles 6 and 1. The yellow color represents significant GO categories that had a P-value of < 0.05. Click here for file

Additional file 5 **Series Test Cluster of Gene Ontology (STC-GO) analysis of profiles 7 and 0**. Additional file 5 contains details of GO based on biological process (BP) enrichment analyses for sets of DE genes of significant cluster profiles 7 and 0. The yellow color represents significant GO categories that had a P-value of < 0.05. Click here for file
