# Supplementary material for: Aberrant host immune response induced by highly virulent PRRSV identified by digital gene expression tag profiling
Source: BMC Genomics. 2010 Oct 7;11:544. doi: 10.1186/1471-2164-11-544 (PMC3091693; doi:10.1186/1471-2164-11-544)
Supplement: Additional file 1 — Seven supplementary figures and one supplementary table. Additional file 1 contains seven supplementary figures and supplementary table 1 in PDF format. Figure S1. Saturation of DGE libraries. Figure S2. The positions of tags in the gene. Figure S3. Effect of library size on the number of genes identified. Figure S4. STC (Series Test of Cluster) analysis of DE genes. Figure S5. Biological process GO terms of profiles 6 and 1. Figure S6. Biological process GO terms of profiles 7 and 0. Figure S7. Differential expression of heat shock genes. Table S1. Tissue distribution of H-PRRSV in infected pigs using QPCR assays. [file 1471-2164-11-544-S1.PDF]

Additional file 1

Seven Supplementary Figures

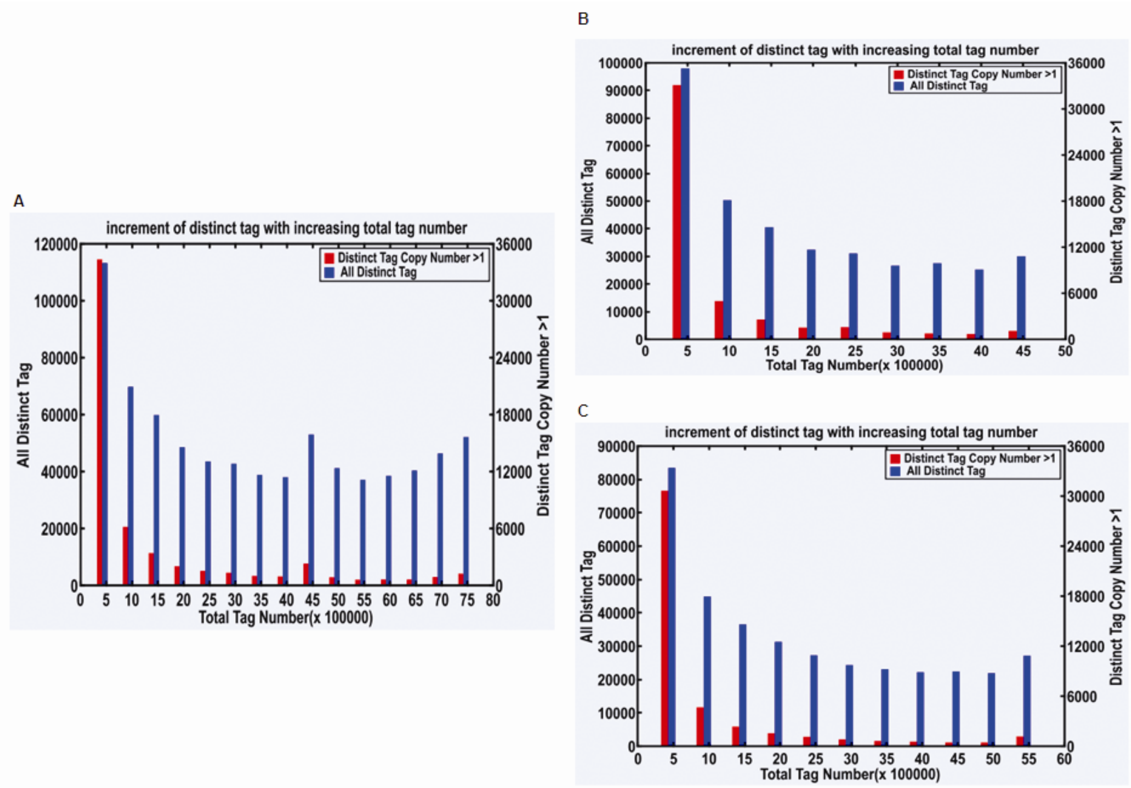

Figure S1. Saturation of DGE libraries. Saturation analysis of the capacity of libraries demonstrated that newly emerging distinct tags were gradually reduced with increase in total sequence tags when the number of sequencing tags was large enough. (A) C; (B) H96; (C) H168

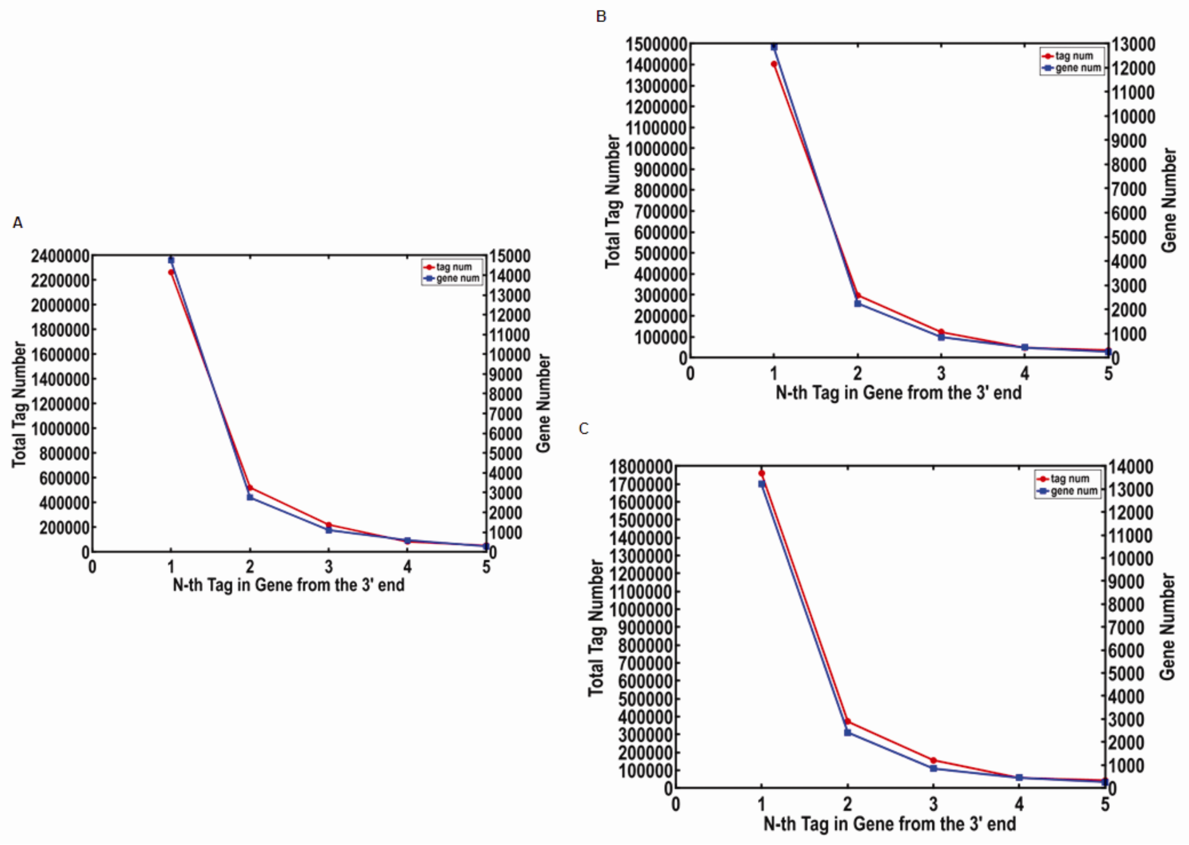

Figure S2. The positions of tags in the gene. Ideally the tag is the closest 3’ end. For alternative splicing or incomplete enzyme digestion, the tag may be the 2nd or 3rd along from the one closest to the 3’ end. (A) C; (B) H96; (C) H168

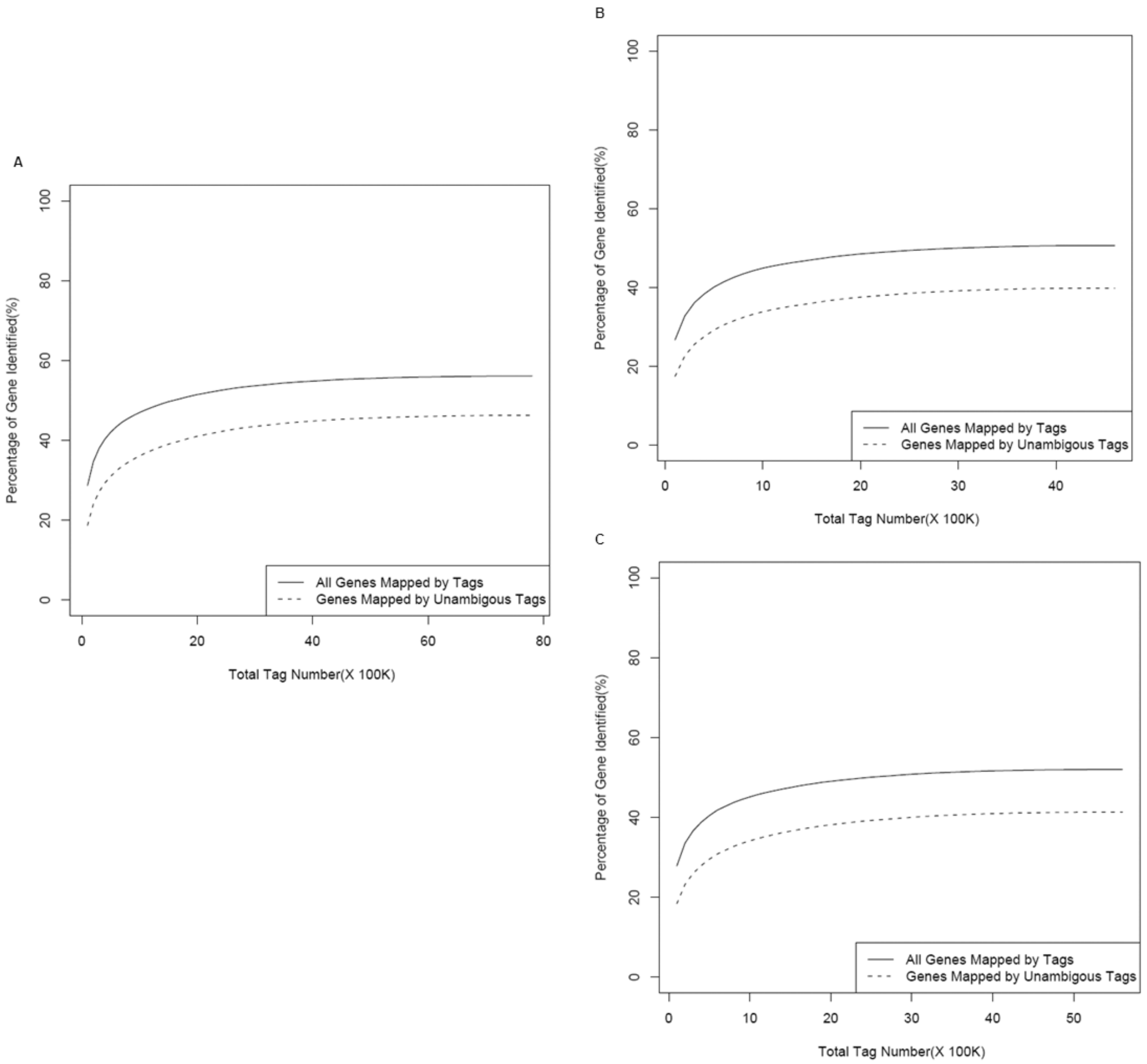

Figure S3. Effect of library size on the number of gene identified. The rate of increase of all genes identified and genes identified by unambigous tags declined as the size of the library increased. When the library size reached two million, we could identify 45% and 35% of all genes and genes identified by unambigous tags, respectively. At this time, library capacity approached saturation. (A) C; (B) H96; (C) H168

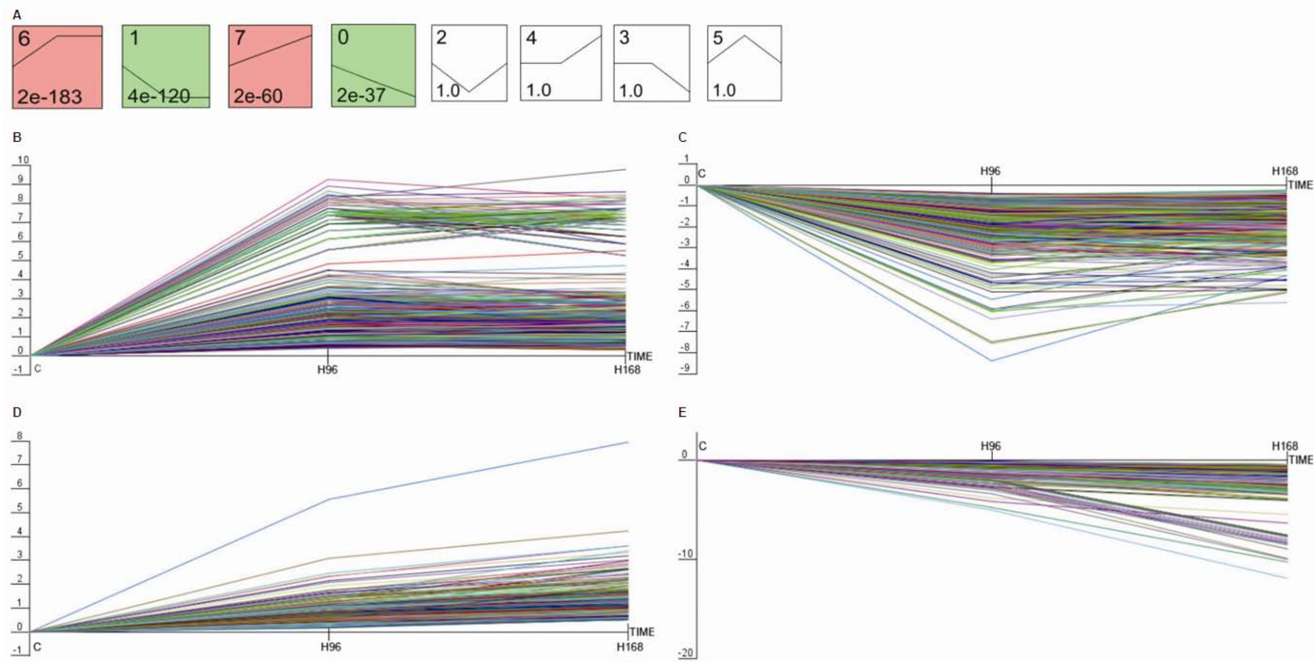

Figure S4. STC (Series Test of Cluster) analysis of DE genes. Dynamic gene expression profiles in 4518 DE genes are shown for eight clusters (A). The eight profiles were ordered on the basis of the *p* value significance of number of genes assigned versus expected. The upper left represents the serial number of the cluster, and the lower left represents the *p* value. (B-E) four significant cluster profiles that have significantly more genes assigned under the true ordering of time points compared to the average number assigned to the model profile in the permutation runs. Y axis indicates the relative gene expression change presented as log2 ratio between UNC lung and H-PRRSV infected lungs at the indicated time points. (B) profile 6 (0,1,1), 726.7 genes were expected but 1519.0 were assigned, p-value = 1.7E-183; (C) profile 1 (0,-1,-1), 454.5 genes were expected but 989.0 were assigned, p-value = 3.8E-120; (D) profile 7 (0,1,2), 324.8 genes were expected but 644.0 were assigned, p-value = 1.9E-60; (E) profile 0 (0,-1,-2), 324.8 genes were expected but 568.0 were assigned, p-value = 1.7E-37.

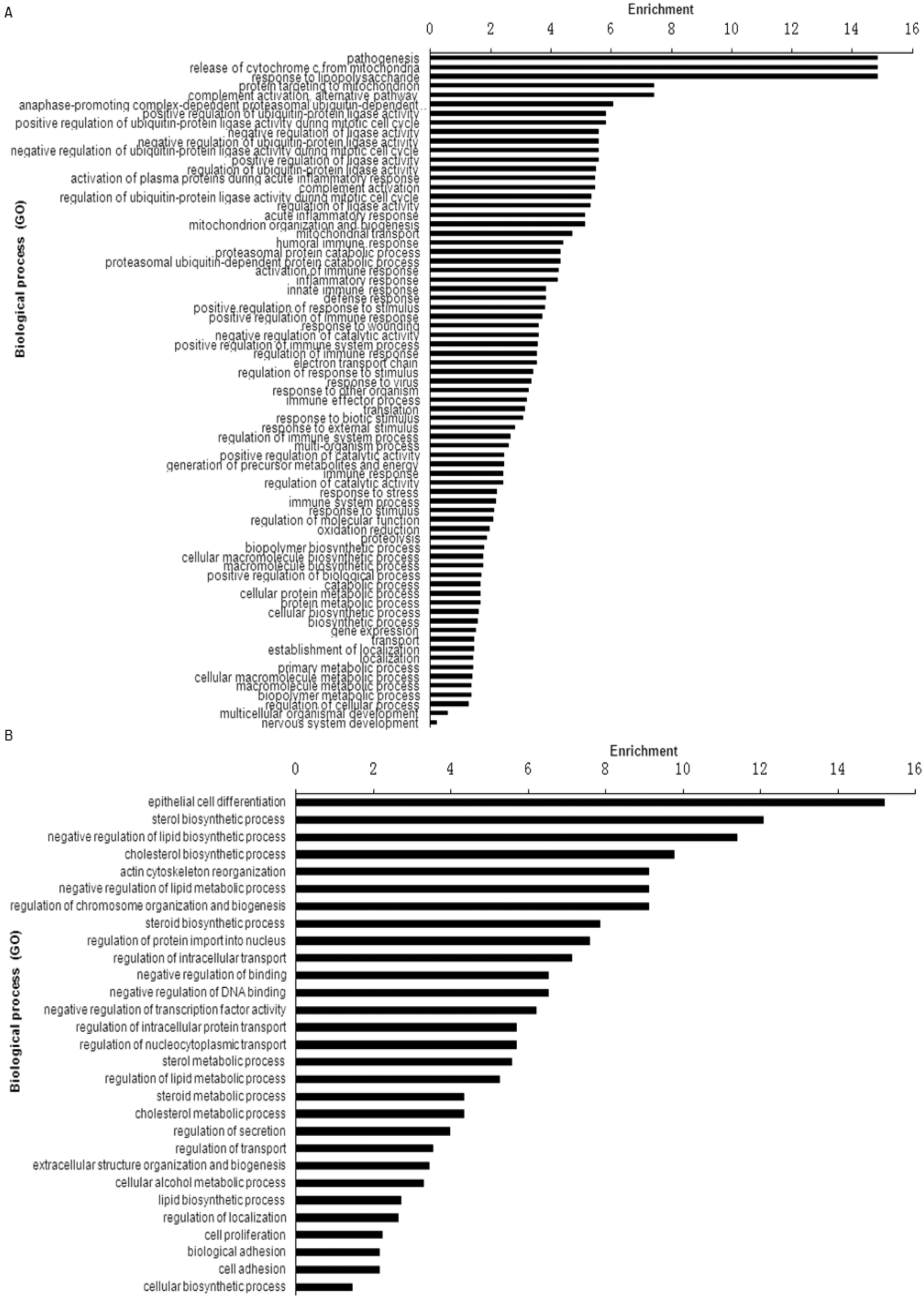
A
Enrichment
0
2
4
6
8
10
12
14
16
Biological process (GO)
pathogenesis
release of cytochrome c from mitochondria
response to lipopolysaccharide
protein targeting to mitochondrion
complement activation, alternative pathway
anaphase-promoting complex-dependent proteasomal ubiquitin-dependent…
positive regulation of ubiquitin-protein ligase activity
positive regulation of ubiquitin-protein ligase activity during mitotic cell cycle
negative regulation of ligase activity
negative regulation of ubiquitin-protein ligase activity
negative regulation of ubiquitin-protein ligase activity during mitotic cell cycle
positive regulation of ligase activity
regulation of ubiquitin-protein ligase activity
activation of plasma proteins during acute inflammatory response
complement activation
regulation of ubiquitin-protein ligase activity during mitotic cell cycle
regulation of ligase activity
acute inflammatory response
mitochondrion organization and biogenesis
mitochondrial transport
humoral immune response
proteasomal protein catabolic process
proteasomal ubiquitin-dependent protein catabolic process
activation of immune response
inflammatory response
innate immune response
defense response
positive regulation of response to stimulus
positive regulation of immune response
response to wounding
negative regulation of catalytic activity
positive regulation of immune system process
regulation of immune response
electron transport chain
regulation of response to stimulus
response to virus
response to other organism
immune effector process
translation
response to biotic stimulus
response to external stimulus
regulation of immune system process
multi-organism process
positive regulation of catalytic activity
generation of precursor metabolites and energy
immune response
regulation of catalytic activity
response to stress
immune system process
response to stimulus
regulation of molecular function
oxidation reduction
proteolysis
biopolymer biosynthetic process
cellular macromolecule biosynthetic process
macromolecule biosynthetic process
positive regulation of biological process
catabolic process
cellular protein metabolic process
protein metabolic process
cellular biosynthetic process
biosynthetic process
gene expression
transport
establishment of localization
localization
primary metabolic process
cellular macromolecule metabolic process
macromolecule metabolic process
biopolymer metabolic process
regulation of cellular process
multicellular organismal development
nervous system development
B
Enrichment
0
2
4
6
8
10
12
14
16
Biological process (GO)
epithelial cell differentiation
sterol biosynthetic process
negative regulation of lipid biosynthetic process
cholesterol biosynthetic process
actin cytoskeleton reorganization
negative regulation of lipid metabolic process
regulation of chromosome organization and biogenesis
steroid biosynthetic process
regulation of protein import into nucleus
regulation of intracellular transport
negative regulation of binding
negative regulation of DNA binding
negative regulation of transcription factor activity
regulation of intracellular protein transport
regulation of nucleocytoplasmic transport
sterol metabolic process
regulation of lipid metabolic process
steroid metabolic process
cholesterol metabolic process
regulation of secretion
regulation of transport
extracellular structure organization and biogenesis
cellular alcohol metabolic process
lipid biosynthetic process
regulation of localization
cell proliferation
biological adhesion
cell adhesion
cellular biosynthetic process

Figure S5. Biological process GO terms of profile 6 and 1. Functional classification of the DE genes was performed according to GO biological processes. A P-value of <0.05 in the two-side Fisher's exact test were selected as significant. DE genes were sorted by the enrichment of GO categories. (A) the biological process GO categores targeted by the DE genes in profile 6; (B) the biological process GO categores targeted by the DE genes in profile 1. The vertical axis is the GO category and the horizontal axis is the enrichment of GO.

A

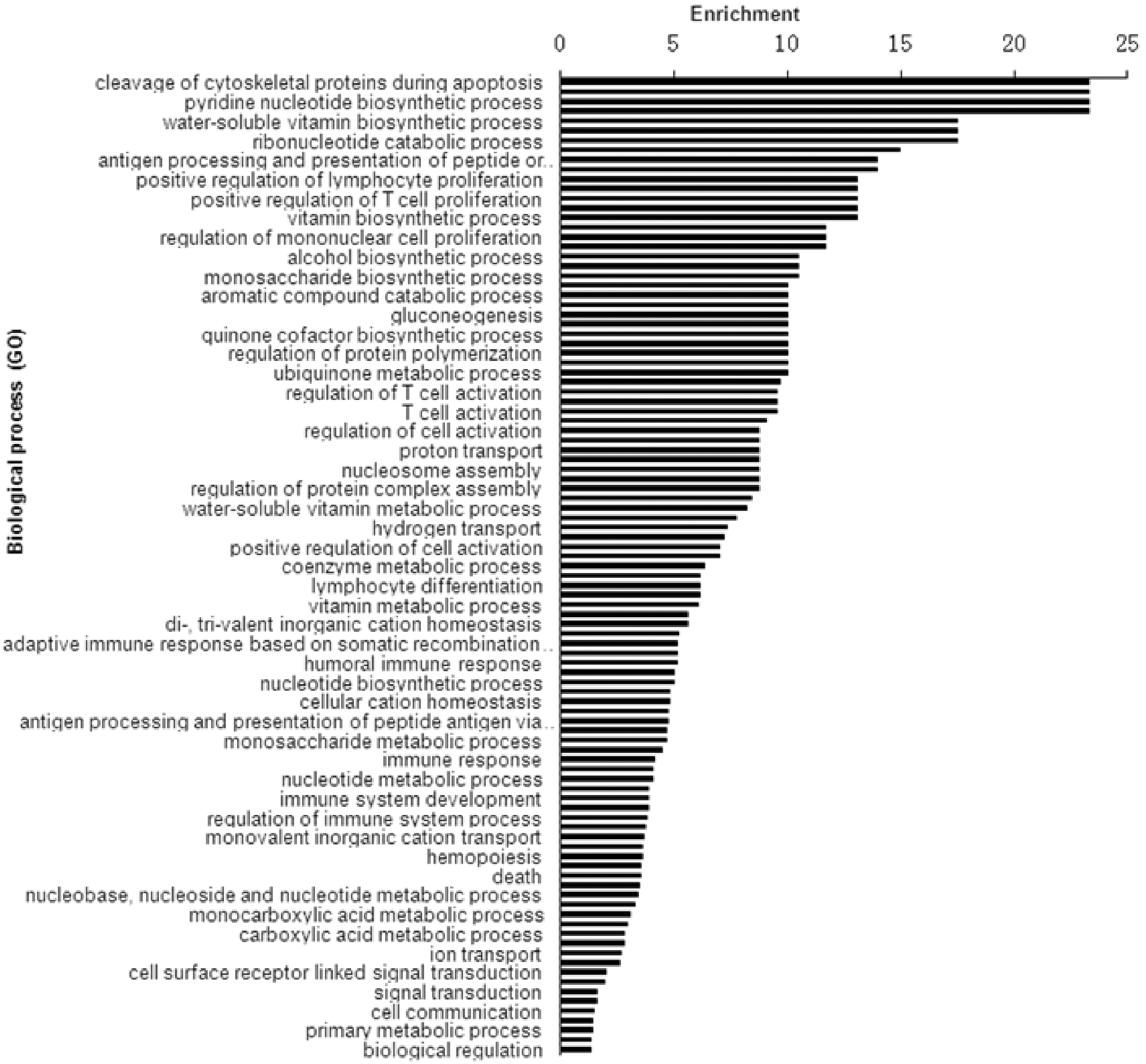

Enrichment
0
5
10
15
20
25
Biological process (GO)
cleavage of cytoskeletal proteins during apoptosis
pyridine nucleotide biosynthetic process
water-soluble vitamin biosynthetic process
ribonucleotide catabolic process
antigen processing and presentation of peptide or..
positive regulation of lymphocyte proliferation
positive regulation of T cell proliferation
vitamin biosynthetic process
regulation of mononuclear cell proliferation
alcohol biosynthetic process
monosaccharide biosynthetic process
aromatic compound catabolic process
gluconeogenesis
quinone cofactor biosynthetic process
regulation of protein polymerization
ubiquinone metabolic process
regulation of T cell activation
T cell activation
regulation of cell activation
proton transport
nucleosome assembly
regulation of protein complex assembly
water-soluble vitamin metabolic process
hydrogen transport
positive regulation of cell activation
coenzyme metabolic process
lymphocyte differentiation
vitamin metabolic process
di-, tri-valent inorganic cation homeostasis
adaptive immune response based on somatic recombination..
humoral immune response
nucleotide biosynthetic process
cellular cation homeostasis
antigen processing and presentation of peptide antigen via..
monosaccharide metabolic process
immune response
nucleotide metabolic process
immune system development
regulation of immune system process
monovalent inorganic cation transport
hemopoiesis
death
nucleobase, nucleoside and nucleotide metabolic process
monocarboxylic acid metabolic process
carboxylic acid metabolic process
ion transport
cell surface receptor linked signal transduction
signal transduction
cell communication
primary metabolic process
biological regulation

B

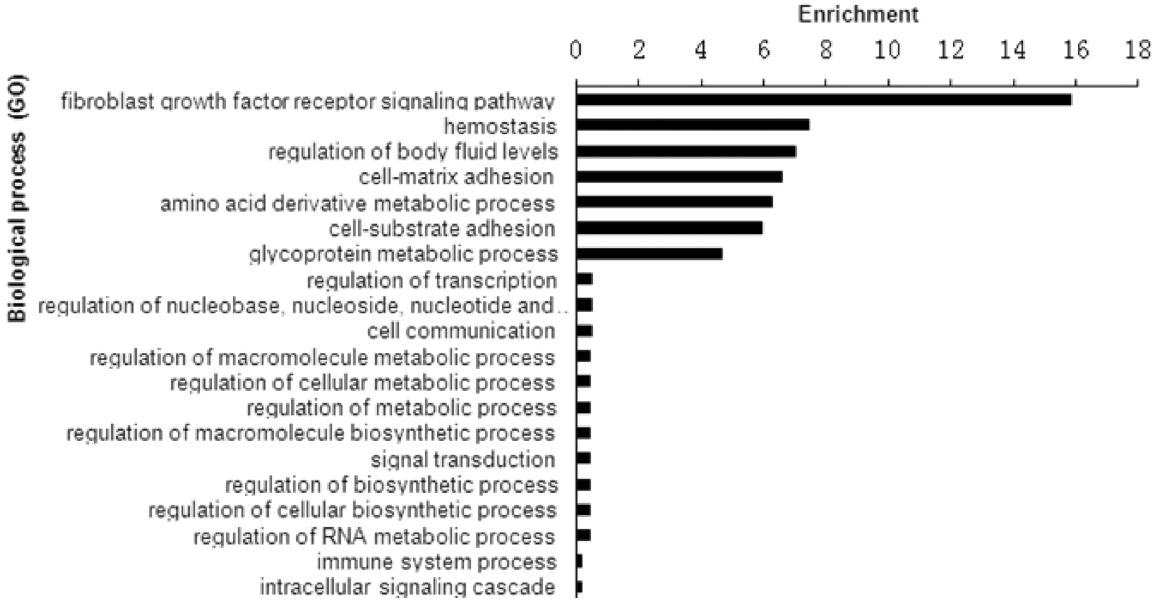

Enrichment
0
2
4
6
8
10
12
14
16
18
Biological process (GO)
fibroblast growth factor receptor signaling pathway
hemostasis
regulation of body fluid levels
cell-matrix adhesion
amino acid derivative metabolic process
cell-substrate adhesion
glycoprotein metabolic process
regulation of transcription
regulation of nucleobase, nucleoside, nucleotide and..
cell communication
regulation of macromolecule metabolic process
regulation of cellular metabolic process
regulation of metabolic process
regulation of macromolecule biosynthetic process
signal transduction
regulation of biosynthetic process
regulation of cellular biosynthetic process
regulation of RNA metabolic process
immune system process
intracellular signaling cascade

Figure S6. Biological process GO terms of profile 7 and 0. Functional classification of the DE genes was performed according to GO biological processes. A P-value of <0.05 in the two-side Fisher's exact test was selected as the significance criterion. These DE genes were sorted by the enrichment of GO categories. (A) the biological process GO categores targeted by the DE genes in profile 7; (B) the biological process GO categores targeted by the DE genes in profile 0. The vertical axis is the GO category and the horizontal axis is the enrichment of GO.

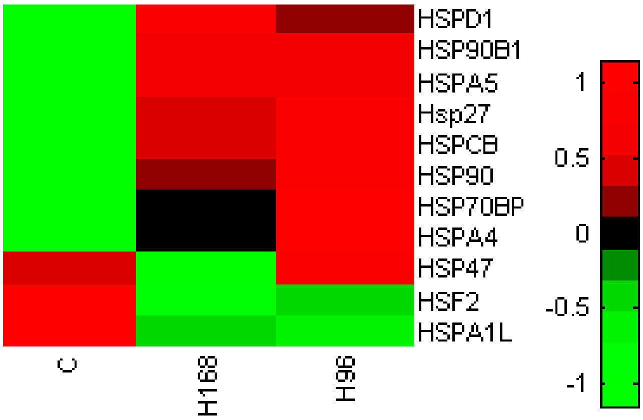

Figure S7. Differential expression of heat shock genes. The red and green colours represent significantly induced or repressed gene expression, respectively. See supplementary Table S2 for full gene names.

Supplementary Tables

Table S1. Tissue distribution of H-PRRSV in infected pigs by QPCR assays

	Ct values[a] ±S.D.[b]		
	C	H96	H168
Serum	None	21.1139±1.1390	20.8849±2.6263
Lung	None	27.4856±2.0069	27.1000±2.0042
Spleen	None	27.5144±0.7899	29.7356±0.1389
Lymph node	None	28.2289±2.4771	30.2200±1.7345
Liver	None	30.2656±0.8086	32.5111±2.5058
Kidney	None	32.6833±1.5282	31.9778±2.8607
Brain	None	33.0167±0.4206	32.4144±1.1881
Heart	None	34.3867±2.0215	34.8667±2.5924

[a]Ct values: Cycle threshold value.

[b]S.D.: Standard deviation.
